# Assessment of genetic diversity and distribution of endophytic fungal communities of *Alternaria solani* isolates associated with the dominant Karanja plants in Sanganer Region of Rajasthan

**DOI:** 10.1186/2193-1801-2-313

**Published:** 2013-07-13

**Authors:** Kartikeya Tiwari, Manish Chittora

**Affiliations:** Microbial Biotechnology Laboratory, Jaipur National University, Jaipur, 302017 Rajasthan India

**Keywords:** *Alternaria solani*, RAPD, Genetic Diversity, Endophytes, NTSYS

## Abstract

Higher plants are ubiquitously colonized with fungal endophytes that often lack readily detectable structures. Current study examines the distribution of endophytic fungal communities within Karanja plants and diversity of novel fungal endophyte *Alternaria solani* isolates collected from different locations of Sanganer region of Rajasthan. Results confirmed that *A. solani* is a major fungal endophyte consortium associated with Karanja plants. PCR Amplified fragments using random amplified polymorphic DNA (RAPD) primers were subjected to unweighted pair group method analysis (UPGMA), which clearly distinguished twelve ecologically diverse *A. solani* isolates. A total of 58 RAPD loci were amplified, out of which 35 (60.34%) were polymorphic and 23 were monomorphic (39.66%) in nature. These polymorphic loci were identified with an average of 2.92 bands per primer. The efficacy of RAPD markers proved as an efficient marker system with respect to detection of polymorphism and number of loci scored and can be used for the identification of a particular isolates, thereby defining core collections and strengthening their exploitation in acquiring novel products produced by them.

## Introduction

Scarcity of natural resources is one of the major issues for the developing countries. Environmental degradation, loss of biodiversity and spoilage of land provide positive contribution to the above. Insufficiency of fuel is a big problem for the burgeoning human population in the world. To overcome this hitch, people are looking for some innovative alternatives. In the last few years, biofuel is emerging as a potent fuel alternate to overcome the fuel scarcity (Strobel & Daisy 2003). For a truly renewable source, crops or other similar agricultural sources would have to be considered.

Rajasthan is one of the twenty five hot spots of global biodiversity and offers most suitable climate for the growth of diverse group of plants. *Pongamia pinnata* L. commonly known as Karanja (Family- Leguminaceae) is one of the important plant of high commercial value. It has been recognized as a major biodiesel producer in India (Tiwari et al. 2010). The tree attains a height of 7–10 meter. The plant is mainly valued for their seeds which contain 30-40% oil. This plant accommodates a large number of fungal endophytes (Saikkonen et al. 2004). It has recently surged that the xerophytic conditions of Sanganer region of Rajasthan accommodate a large number of fungal endophytes (Lucero et al. 2006), which led to a considerable amount of research regarding the role of these fungi in host plants (Frohlich et al. 2000Strobel 2003; Tan & Zou , Tan & Zou Tan & Zou 2001Tejesvi et al. 2007Zou et al. 2000).

Endophytes, microorganisms that reside in the tissues of living plants are relatively unstudied and potential sources of novel natural products for exploitation in medicine, agriculture and industry (Schulz et al. 2002). It is noteworthy that, of the nearly 300,000 plant species that exist on the earth, each individual plant is host to one or more endophytes. Fungal endophytes with the potential of biofuel production/bioactive compounds can be harvested from these plants. This fact alone helps in the elimination of the problem of biofuel scarcity. Due to extraordinary role of these fungi in ecosystem, it is necessary to explore them at morphological, ecological and molecular level.

It seems obvious that endophytes are a rich and reliable source of genetic diversity. Characterization of genetic diversity is a prerequisite for efficient conservation and utilization of genetic resources. Conventionally, fungal species have been characterized based on morphological characteristics. During the last few decades, several PCR based molecular marker techniques such as random amplified polymorphic DNA (RAPD) (Saikkonen et al. 2004), inter simple sequence repeat (ISSR) (Zietkiewicz et al. 1994), simple sequence repeat (SSR) (Litt & Luty 1989), Restriction fragment length polymorphism (RFLP) (Botstein et al. 1980), Amplified fragment length polymorphism (AFLP) (Vos et al. 1995) etc. have been developed and establish wide application in the assessment of genetic diversity (Haugland et al. 2004; Sette et al. 2006).

Among them, RAPD markers have caught the fancy of many individuals in the field of applied fungal taxonomy due to their low cost and easy to handle (Guo et al. 2001; &^2003^Hawsksworth 1991Williams et al. 1990). These markers are amplification products of anonymous sequences using single, short and arbitrary oligonucleotide primers (8–10 bp). DNA sequence difference between individuals in a primer-binding site may results in the failure of the primer to bind and hence in the absence of a particular band among amplification products. RAPD markers provide a quick and efficient screening of DNA sequence as they require small amount of DNA, involve non-radioactive assay, need a simple experimental set-up, do not require species specific probe libraries or hybridization step.

In the present study, assessment of genetic diversity among fungal endophytes is a vital part because it explores the endophytic association, distribution and host specificity between *P*. *pinnata* and novel fungal endophytes. This study will help in resolving genetic relationship among different isolates and can be used for selection of desired isolate for domestication.

## Materials and methods

### Site description

**Figure 1:**
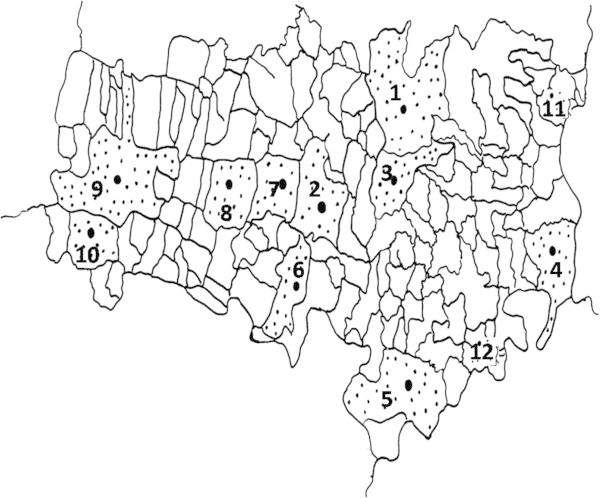
**Map of Sanganer region of Rajasthan shaded portion showing sampling sites.**

**Figure 2:**
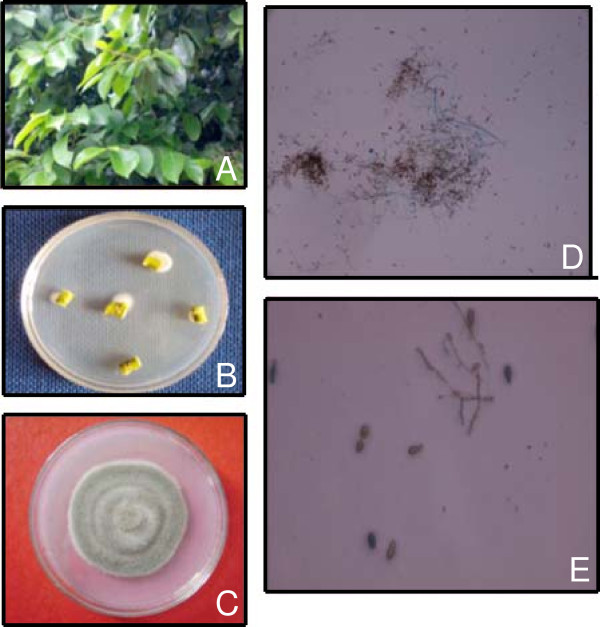
**Methodology of endophytic fungal isolation from Karanja plants. (A)** Mature plant of *Pongamia pinnata* L. **(B)** Sporulating fungal endophytes from nodal explants. **(C)** Pure culture of *A*. *solani* growing on PDA media. **(D)** Conidial development of *A*. *solani*. **(E)** Mature conidia of *A*. *solani* with cross and longitudinal septa.

The samples were collected from 12 different regions of Sanganer (Table 1, Figure 1). These sites were almost similar with respect to their climatic conditions and provide optimum growth conditions to the plant *P. pinnata* (Figure 2A).

**Table 1:** **Details of*Alternaria solani*isolates collected from various sites of Sanganer region**

Accession number	Collection site
1	Khonagorion
2	Muhana
3	Jagatpura
4	Goner
5	Powlia
6	Watika
7	Kalwara
8	Neola
9	Bagru
10	Rampura
11	Bassi
12	Chaksu

### Sample collection

Plants samples (*P. pinnata*) from the entire twelve sites of Sanganer region, especially those with an unusual biology and possessing novel strategies for survival were selected for the study (Strobel & Daisy 2003). The samples were collected in sterilized polythene bags, during the month of July- November.

### Isolation of fungal endophytes

The leaves, nodes and internodes were used as explants for isolation of fungal endophytes (Figure 2B). All explants were surface-sterilized by dipping in 75% ethanol for 1 minute, 4% sodium hypochlorite for 5 minutes followed by rinsing three times in sterilized double distilled water (Frohlich et al. 2000). In each Petri dish (9 cm diameter), a total of four-five processed explants were evenly spaced onto the surface of Potato Dextrose Agar (PDA) media supplemented with 200 μg/ml tetracycline. Cultures were incubated at 28°C and observation was recorded regularly. The sporulating mycelia of fungi appeared on the plates were carefully isolated, sub-cultured and pure culture were maintained (Nagamani et al. 2006) (Figure 2C).

### Identification of fungal endophytes

The isolated fungal endophytes were identified on the basis of morphological features like colony characterization, growth on different media, color of colony (front and reserve), conidial development, size, shape and attachment of conidia (Nagamani et al. 2006). Then the fungus was grown in a slide culture by which the pores of the fungus remain undisturbed and attached to the sporophores thus facilitating in identification (Anthony & Walkes 1962). This technique was performed for the examination of various stages of conidia formation and proper identification of the sporulating fungi (Promputtha et al. 2005). The microscopic identification of fungal endophytes was carried out by lacto phenol cotton blue staining method (Nagamani et al. 2006) (Figure 2D-E).

### Frequency distribution and colonization rate

Data analysis was carried out on the basis of Colonization rate (%) of fungal endophytes which was equal to the number of segments colonized by a single endophyte divided by the total number of segments observed X 100 (Raviraja 2005Raviraja et al. 2006).

### Molecular analysis

Assessment of genetic diversity among 12 fungal isolates of *A*. *solani* isolated from different ecological niche were carried out using PCR based RAPD primers.

### Isolation of DNA

The fungal DNA was isolated as per the standardized protocol (Guo et al. 2003 & 2004).

### Purification of DNA samples using RNase treatment

The isolated DNA sample was raised up to 400 μl with TE buffer. Two μl of RNase A (10 mg/ml) was added, mixed by inversion and incubated at 37°C for 30 minutes. DNA was precipitated using 50 μl of 4 M ammonium acetate and 950 μl of pre chilled ethanol. The content was mixed gently by inversion and spin at 10, 000 rpm for 5 minutes. The supernatant was discarded and the pellet was dried by inverting the tube on the paper towel for 15 minutes. DNA pellet was dissolved in 40 μl of TE buffer and stored at 4°C.

### RAPD primers

Fifty RAPD primers were initially screened for the PCR amplification of the genomic DNA isolated from the *A*. *solani*. Out of which 12 primers gave good amplification in terms of number of bands and reproducibility (Table 2).

**Table 2:** **Primer-wise score of PCR amplification products scored in the 12 isolates of*Alternaria solani***

Primer	Number of PCR amplification fragments generated	
	Monomorphic bands	Polymorphic bands
GCC-4	5	3
GCC-7	7	2
GCC-14	2	1
GCC-16	5	4
GCC-19	3	5
GCC-20	6	2
GCC-24	4	3
GCC-47	8	3
GCC-49	6	2
GCC-74	5	4
GCC-89	3	4
GCC-91	4	2

### PCR amplification conditions

Each 25 μl reaction mixture contained 12.5 μl of 2 X PCR buffer, 2 μl each of dATP, dGTP, dTTP and dCTP, 1U Taq DNA polymerase, 25 pmol random decamer primer and 50 ng of genomic DNA for PCR amplification.

### Data analysis

Amplification products were scored from the gel images as presence or absence of bands. Each band was treated as one marker. Homology of bands was based on the distance of migration of amplified DNA fragments according to their molecular weights in the gel. The presence of band 0percentage of the total number of bands produced in fingerprinting profiles. Cluster analysis for the genetic distance was then carried out using UPGMA (Unweighted Pair Group Method Analysis) clustering method (Crous et al. 2006Tedersoo et al. 2006). The genetic distances obtained from cluster analysis through UPGMA were used to construct the dendrogram, depicting the relationships of the clones using computer program NTSYS pc version 2.02 (Rohlf 1997).

## Results

**Figure 3:**
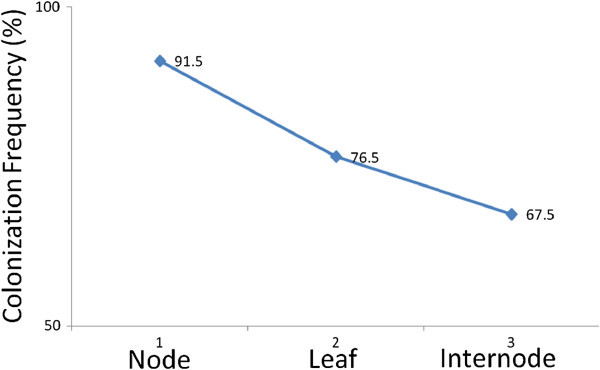
**The colonization frequency (CF) of fungal endophytes in explants (node, leaf and internode) of*Pongamia pinnata*L.**

**Figure 4:**
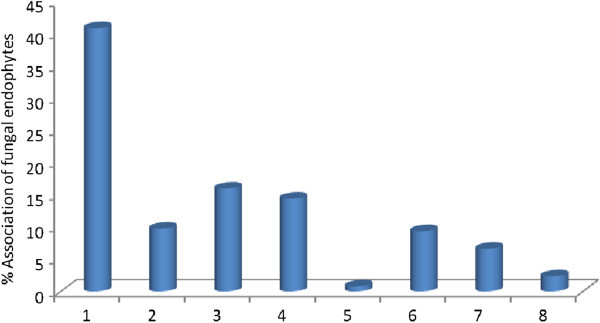
**Fungal endophytes isolated from plant*Pongamia pinnata*L.** (1) *Alternaria solani* (2) *Curvularia lunata* (3) *Fusarium oxysporum*. (4) *Colletotrichum gleosporioides* (5) *Cladosporium cladosporioides*. (6) *Helminthosporium populosum* (7) *Aspergillus flavus* (8) *Phomopsis viticola.*

Maximum number of fungal endophytes were isolated from nodal explants of *P*. *pinnata* (CF=91.5%) as compared to leafy explants (CF=76.5%) and internodal explants (CF=67.5%) (Figure 3). A total of 8 different endophytic fungal genera were isolated from nodal explants. Among these eight isolates, *A*. *solani* showed highest occurrence (40.85%) followed by *Fusarium oxysporum* (15.95%) and *Colletotrichum gleospoirioides* (14.39%). In contrast, *Curvularia lunata* (9.72%), *Helminthosporium papulosum* (9.33%), *Aspergillus flavus* (6.61%), *Phomopsis viticola* (2.33%) and *Cladosporium cladosporioides* (0.77%) were showed low frequency of colonization (Figure 4). The difference in endophyte assemblages from various tissues indicated that some individual dominant endophytic fungal taxa have an affinity for different tissue types and this might reflect their potential for utilizing a specific substrate in connection with the location of the plant sample (Arnold & Lutzoni [Bibr CR2][Bibr CR6]).

**Figure 5:**
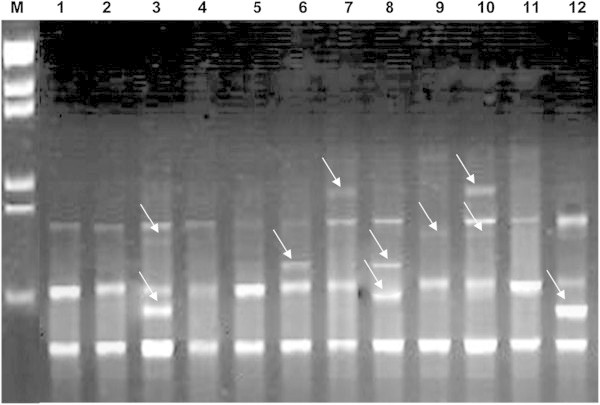
**RAPD profile of twelve isolates of*Alternaria solani*amplified using RAPD primer GCC-19.**

Since, *A*. *solani* showed dominant endophytic association as compared to other endophytic fungal communities. A total of 12 different *A*. *solani* isolates were collected from different regions of Sanganer (Rajasthan) and further investigated for their genetic diversity in nature. These isolates were amplified using 50 random decamer primers to ascertain the level of genetic diversity among them. Of the 50 primers screened, 12 primers produced reproducible results. A total of 58 RAPD loci were amplified from different isolates. Most of the PCR products were in size range of 100–2000 bp with 4.83 bands per RAPD primer. Of the 58 bands scored 35 (60.34%) were found to be polymorphic (either occurring in or absent in less than 95% of all isolates) and 23 (39.66%) were found to be monomorphic in nature (Table 3). A total of 35 polymorphic loci were identified with an average of 2.92 bands per primer. The frequencies of polymorphic bands obtained varied from primer to primer. Wide genetic variation between isolates of the species was evident from the high number of polymorphic marker and unique bands, even though small number of isolates available (Figure 5).

**Table 3:** Distribution of amplified fragments in*Alternaria solani*isolates*

Parameters	Values
Total number of primers screened	50
Number of primers showed amplification	18
Number of primers producing polymorphism	12
Total number of loci scored	58
Total number of polymorphic loci	35
Size of amplified bands	100-2000 bp
Average number of bands per primer	4.83
Average number of polymorphic bands per primer	2.92
% bands which are polymorphic	60.34

*Data pooled from studies on 12 isolates of *Alternaria solani*.

**Figure 6:**
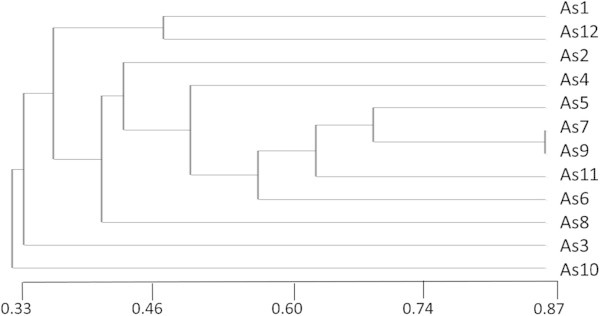
**Dendrogram showing UPGMA clustering of 12 isolates of*A.solani.***

Data obtained from RAPD analysis alienated 12 isolates into 3 large cluster groups (Figure 6). The first group is further divided into 2 subgroups (A & B). Sub group A has 2 isolates (As1 and As12) whereas, subgroup B contain 8 isolates (As2, As4, As5, As7, As9, As11, As6 and As8). Group second and third contain only one isolate As3 and As10, respectively.

## Discussion

The mechanism through which endophytes exists and respond to their surrounding must be better understood in order to be more predictive about which higher plants to seek and spend time in isolating microfloral compounds. This may facilitate the product discovery processes. Certainly, one of the major problems facing the future of endophyte biology and natural-product discovery is the rapid diminishment of rainforests, which holds the greatest possible resource for acquiring novel microorganisms and their products.

Endophytic fungal communities from different habitats are indeed different: communities associated with Sanganer region of Rajasthan are poorly investigated group of microorganisms that represents an abundant and dependable source of bioactive and chemically novel compounds with potential for exploitation in a wide variety of medical, agriculture and industrial arenas (Strobel & Daisy 2003). Endophytes belonging to potentially pathogenic species were also isolated. Hence, it would appear that certain fungal species, which are notoriously pathogenic in certain plant types, might behave as endophytes in others, thereby not inducing disease symptoms. In the above investigation, the most frequently isolated endophytic fungal species was *A*. *solani*, dominantly associated with the Karanja plants.

These molecular markers can demonstrate similarities and dissimilarities between different isolates of same species even when a morphological description is severely limited. Among them, RAPD despite having certain disadvantages (dominant nature and stringent optimization of assay), can produce multilocus profiles, widely spanning the genome even in the absence of any prior genetic/sequence information. Present study provides a molecular profile based on RAPD marker, which can be used for the identification of a particular *A*. *solani* isolate, thereby defining their core collection and strengthening their exploitation in acquiring novel products produced by them.
